# Biodegradation of polycyclic aromatic hydrocarbons by a bacterial consortium enriched from mangrove sediments

**DOI:** 10.1186/s40201-014-0114-6

**Published:** 2014-08-21

**Authors:** Mohsen Shahriari Moghadam, Gholamhossein Ebrahimipour, Behrooz Abtahi, Alireza Ghassempour, Mehri Seyed Hashtroudi

**Affiliations:** Department of Marine Biology, Faculty of Biological Science, Shahid Beheshti University, G.C, Tehran, Iran; Department of Environment, Faculty of Natural Resources, University of Zabol, Zabol, Iran; Department of microbiology, Faculty of Biological Science, Shahid Beheshti University, G.C, Tehran, Iran; Department of Phytochemistry, Medicinal Plants and Drugs Research Institute, Shahid Beheshti University, G.C, Tehran, Iran; Iranian National Institute for Oceanography (INIO), Tehran, Iran

**Keywords:** Bioremediation, Taguchi experimental design, Soil contamination, PAHs

## Abstract

Polycyclic aromatic hydrocarbons (PAHs) biodegradation in contaminated sediment is an attractive remediation technique and its success depends on the optimal condition for the PAH-degrading isolates. The aims of the current study was to isolate and identify PAHs-degrading bacteria from surface sediments of Nayband Bay and to evaluate the efficiency of statistically based experimental design for the optimization of phenanthrene (Phe) and Fluorene (Flu) biodegradation performed by enriched consortium. PAHs degrading bacteria were isolated from surface sediments. Purified strains were then identified by 16S rDNA gene sequence analysis. Taguchi L_16_ (4^5^) was employed to evaluate the optimum biodegradation of Phe and Flu by the enriched consortium. Total of six gram-negative bacterial strains including *Marinobacter hydrocarbonoclasticus*, *Roseovarius pacificus*, *Pseudidiomarina sediminum* and 3 unidentified strains were isolated from enrichment consortium, using Fluorene (Flu) and phenanthrene (Phe) as the sole carbon and energy source. The enriched consortium showed highest degradation abilities (64.0% Flu and 58.4% Phe degraded in 7 days) in comparison to a single strain cultures or mixtures. Maximum biodegradation efficiency was occur at temperature = 35°C; pH = 8; inoculum size = 0. 4 OD_600nm_; salinity = 40 ppt; C/N ratio = 100:10. In conclusion our results showed that, indigenous bacteria from mangrove surface sediments of Nayband Bay have high potential to degrade Flu and Phe with the best results achieved when enriched consortium was used.

## Introduction

Mangrove forests are among the most productive coastal ecosystems along the coastline of tropical and subtropical regions. Due to their inherent physical and chemical properties (being rich in organic matter as well as anaerobic and reduced condition of soil) mangrove forests have an extraordinary capacity to accumulate materials discharged to the near shore marine environment [1]. Polycyclic aromatic hydrocarbons (PAHs) are aromatic hydrocarbons with two or more fused benzene rings. The US Environmental Protection Agency (EPA) has identified 16 PAHs as particularly important due to their carcinogenic and mutagenic properties [2,3]. Several polluting anthropogenic activities such as oil spilling, incomplete combustion of fossil fuel, ship traffic, urban runoff and industrial activities have led to significant accumulation of PAHs in marine environments especially those near industrial cities [4,5]. In essence it is necessary to remove PAHs from environment and to minimize their adverse effects. Microbial degradation is primary mechanism in elimination of PAHs and xenobiotic substances [6]. Numerous investigators have studied the use of mangrove bacteria for biodegradation of PAHs [7,8]. In practice, the efficiency of PAH biodegradation can be affected by several factors, such as bacterial population size, temperature, pH, nutrient, salinity, etc. which may be optimized to achieve a more efficient process [9,10].

Conventional optimization procedures are performed by altering one parameter at a time and keeping all other parameters at fixed levels. As a result, the impact of that particular parameter on the process can be assessed. However, these procedures are time consuming, require more experimental data sets and cannot provide information about the mutual interactions of the parameters. Recently, the use of an orthogonal array approach called ‘Taguchi method’ has been successfully examined in the field microbiological optimization procedures [11,12] which enables one to examine many factors simultaneously and also provides much quantitative information by doing few experimental trials.

The Nayband Bay, as a sensitive coastal region, provides with some ecologically important habitats such as coral reefs, seagrass beds, and mangrove forests. The intensity of industrialization and urbanization began to increase around the area 15 years ago when the South Pars Gas Complex (SPGC) was established and the Assaluyeh harbor was enlarged. Growing industrial activities in the area and their consequent marine polluting effects has threatened different marine habitats in the Nayband Bay in recent years. In essence, the use of practicable and applicable bioremediation protocols for habitat restoration in the area seems mandatory. However, there is little literature on the biodegrading efficacy of PAHs degrading bacteria in the Iranian coasts of Persian Gulf and careful examinations must be done to optimize their operative efficiency. The present study was conducted (1) to isolate the PAHs-degrading bacteria from mangrove sediments; (2) to assess the biodegradation ability of the enriched bacterial consortium and the isolates and (3) to investigate the effects of various factors on the degradation of fluorene (Flu) and phenanthrene (Phe) by enriched consortium using orthogonal experimental design. The study was conducted at Shahid Beheshti University, Iran during 2012–2013 period.

## Materials and methods

### Collection of sediments

Aerobic surface sediment samples were randomly collected from the mangrove forests at Nayband bay- Iran, during low tides. Sediment samples were mixed thoroughly to form a composite sample and then sealed into aluminum foils. The sample was transferred to the laboratory and stored at 4°C until inoculation time. Physicochemical properties of environmental factors of the sampling area (pH = 8; temperature = 30°C; salinity = 40 ppt) were measured using HQ40d multimeter.

### Chemicals

All solvents (GC gradient grade) and chemicals were purchased from Merck® chemical company. Stock solution of Flu-Phe mix was prepared by dissolving approximately 2 g of each component in 40 ml acetone. The solutions were stored at 4°C for further use.

### Enrichment of PAH-degrading bacterial consortium

In order to enrich PAH-degrading bacterial consortium, predetermined amount of the Flu-Phe mix stock solution was added to a 250-ml conical flask to obtain the final concentration of 1000 mg.L^−1^. After evaporation of the solvent (acetone), the flask was filled with 45 ml sterilized mineral salt medium (MSM) (1.0 g NH_4_Cl, 0.5 g K_2_HPO_4_, 0.01 g FeSO_4_.7H_2_O in 1 L 0.45 μm Millipore-filtered 40 ppt local seawater). Then after, approximately, 5 g of fresh mangrove sediment sample was transferred into the conical flask and adjusted to pH = 8.0. The flask was incubated at 30°C on a rotary shaker at 140 rpm during of which PAH utilization in the enriched cultures was monitored by a decrease in the amount of PAH crystals, color changes in the medium (from pale color to dark red), and by increase in bacterial biomass. Then, 5 ml of the enriched culture was transferred to a fresh medium and incubated under the same conditions. This process was repeated for four times to obtain the enriched PAH degrading consortium.

### Isolation and identification of bacterial strains

At the end of the enrichment process, bacterial strains in the consortium were isolated by spreading the 10-fold serially diluted consortium on MSM agar plates coated with a layer of Flu-Phe mix on the surface or on nutrient agar plates (3 g peptone, 5 g yeast extract, 1.5% agar in 1 L of filtered seawater). Then, Bacterial colonies were picked off from the plates, and purified by repetitive streaking onto nutrient agar plates. Purified strains were then identified by biochemical tests such as Gram staining and oxidation/fermentation tests. Further molecular identification of the strains was performed by 16S rDNA gene sequences analysis. For this purpose, DNAs of the isolated bacteria were extracted using the bacterial DNA extraction kit (Roche®- Germany). The isolated strains were then identified by 16S rDNA gene sequence analysis after amplification of the gene by PCR using the set of primers 27 F (5- AGA GTT TGA TCC TGG CTC AG-3) and 1510R (5-GGT TAC CTT ACG ACT T-3). DNA sequences of the cloned 16S rDNA fragments were compared using BLAST at http://www.ncbi.nlm.nih.gov/BLAST/ maintained by National Center of Biotechnology Information (NCBI).

### Biodegradation of Flu and Phe by enriched consortium and bacterial isolates

Isolated strains as well as enriched consortium were first cultivated on nutrient agar plates and then transferred on to the MSM (1.0 g NH_4_Cl, 0.5 g K_2_HPO_4_, 0.01 g FeSO_4_.7H_2_O in 1 L of 0.45 μm Millipore-filtered seawater) supplemented with 1% (v/v) light crude oil as the sole source of carbon and incubated for 3 days at 30°C, at the end of which the cells were collected by centrifugation at 8000 rpm for 15 min, and washed twice in sterilized sea water. To assess the utilization rates of Flu-Phe mix using the above mentioned bacteria, microbial inoculums with final optical density (OD_600nm_) of 0.15 were passed to the following media: (I) MSM + Flu-Phe mix (II) MSM + Flu-Phe mix + 1 w/w% Tween-80. In order to determine the abiotic losses of Flu-Phe mix, the same MSM media without any microbial inoculum were also retained. The experimental cultures were performed in triplicate at 30°C in the dark at 140 rpm for 10 days. The entire medium for each trial was used for analysis of the Flu and Phe concentrations.

### Optimization of biodegradation ability of enriched consortium

To maximize Flu and Phe degradation, parameter optimization was done using the Taguchi experimental design, L_16_ (4^5^). The different levels of the studied factors and the layout of the L_16_ Taguchi’s orthogonal array are shown in Table 1 and Table 2. During the optimization process, Flu or Phe were used as a sole carbon source and spiked to the 250 ml conical flask at 500 mg.L^−1^ concentrations for each. Distilled water and NH_4_Cl were used to adjust the salinity and C/N ratio, respectively. In order to reach to the initial inoculum sizes determined by Taguchi experimental design, the enriched consortium was first cultured on MSM supplemented with 1% (v/v) light crude oil as the sole source of carbon for 3 days at 30°C. The cells were then collected by centrifugation at 8000 rpm for 15 min, washed twice in sterilized sea water and used in designed trials. The same experimental setup, without bacterial inoculation, was prepared as a control trials for checking abiotic losses of PAHs. The entire medium of each flask was used for analysis of the Flu and Phe concentrations at 3^rd^, 7^th^ and 20^th^ day. All experimental trials (n = 3) were conducted at 140 rpm shaking approach.

**Table 1:** **Combinations of the five factors and four levels on Flu and Phe biodegradation by enriched consortium**

**Factor**	**Level**			
	**I**	**II**	**III**	**IIII**
pH	6.5	7	7.5	8
Temperature (°C)	20	25	30	35
Inoculum size (OD_600nm_)	0.05	0.1	0.2	0.4
Salinity (ppt)	25	30	35	40
Carbon/Nitrogen (molar)	100:5	100:10	100:20	100:40

**Table 2:** **Experimental conditions for Flu and Phe biodegradation based on the orthogonal design form L**_**16**_ **(4** ^**5**^ **) and results of Flu and Phe biodegradation and first order rate constant (k) at the end of 20-day experiments**

**EN**	**pH**	**Tem**	**Sal**	**In**	**C/N**	**Final biodegradation%**		**k of Flu (h-** ^**1**^ **)**	**k of Phe (h-** ^**1**^ **)**	**R** ^**2**^ **Flu**	**R** ^**2**^ **Phe**
						**Flu**	**Phe**				
1	8	20	25	0.05	100:5	16.26 ± 0.29	16.45 ± 3.63	0.008	0.009	0.68	0.71
2	8	25	30	0.1	100:10	66.31 ± 7.02	66.07 ± 7.19	0.052	0.053	0.93	0.91
3	8	30	35	0.2	100:20	76.35 ± 5.09	77.00 ± 3.00	0.070	0.070	0.91	0.90
4	8	35	40	0.4	100:40	92.18 ± 1.51	89.84 ± 3.03	0.122	0.108	0.92	0.91
5	7.5	20	30	0.2	100:40	78.08 ± 5.07	73.88 ± 8.20	0.080	0.072	0.98	0.98
6	7.5	25	25	0.4	100:20	77.83 ± 3.50	80.59 ± 4.51	0.070	0.078	0.92	0.91
7	7.5	30	40	0.05	100:10	85.68 ± 7.55	78.74 ± 4.29	0.103	0.078	0.99	0.96
8	7.5	35	35	0.1	100:5	82.28 ± 6.06	77.13 ± 3.72	0.089	0.075	0.96	0.97
9	7	20	35	0.4	100:10	82.16 ± 6.29	79.28 ± 5.59	0.088	0.080	0.95	0.94
10	7	25	40	0.2	100:5	83.82 ± 4.48	76.53 ± 6.28	0.094	0.075	0.97	0.98
11	7	30	25	0.1	100:40	87.17 ± 4.47	80.61 ± 6.60	0.105	0.084	0.99	0.99
12	7	35	30	0.5	100:20	80.38 ± 5.62	76.42 ± 6.41	0.085	0.076	0.97	0.99
13	6.5	20	40	0.1	100:20	47.33 ± 4.17	46.35 ± 3.80	0.032	0.032	0.96	0.97
14	6.5	25	35	0.05	100:40	42.13 ± 3.18	38.71 ± 5.10	0.026	0.025	0.95	0.98
15	6.5	30	30	0.4	100:5	72.55 ± 6.67	68.68 ± 3.56	0.061	0.056	0.77	0.74
16	6.5	35	25	0.2	100:10	73.89 ± 3.82	69.85 ± 6.52	0.061	0.056	0.69	0.68

EN, Experiment number; Tem, Temperature (°C); Sal, Salinity (ppt); Optical density (OD_600nm_); C/N, Carbon/Nitrogen (molar).

### Extraction and analysis of Flu and Phe

The concentration of Flu and Phe were determined according to Wu et al. [6]. The culture medium was first transferred to a 250 ml pre-ash conical flask by addition of 20 μL m-terphenyl as internal standard (prepared in acetone with concentration of 1000 mg.L^−1^) and 25 ml ethyl acetate and shacked for 15 min. After separation from the water layer, the organic solvent layer was transferred to a clean conical flask before the second extraction by another aliquot of 25 ml ethyl acetate. Finally, two extracts were combined together, dried by anhydrous Na_2_SO_4_ and the volume was adjusted to 50 ml. About 1 ml of the extract sample was transferred into a 1.5 ml brown vial and analyzed by GC-FID .The GC-FID was equipped with a HP-5MS fused silica capillary column (60 m × 0.25 mm ID × 0.25 lm thickness, Agilent Technologies, USA) with the injector and detector temperature of 280°C and 300°C, respectively. Nitrogen was used as the carrier gas. The oven temperature program was set as follows: The oven temperature program was from 80°C (for 2 min) to 120°C at a rate of 10°C/min and from 120°C to 300°C at a rate of 4°C/minute and held at 300°C for 15 min. The identification and quantification of chemicals were conducted based on matching their retention times of standards [11].

### Biodegradation kinetics of fluorene and phenanthrene

The biodegradation kinetics of Phe and Flu were described using the first order rate model:

$$ C={C}_0{e}^{- kt} $$

where C is the Phe concentration in the medium at time t, C_0_ is the initial Phe concentration and k is the first order rate constant of reaction.

### Biodegradation of Flu and Phe by enriched consortium under different conditions

Based on the optimal conditions detected from the biodegradation percentages at the end of experiment and first order rate constant (k) of Phe and Flu biodegradation, the consortium was inoculated into sterile 250 ml flasks containing 50 ml of MSM containing 1000 mg.L^−1^ concentrations of Flu and Phe and residual PAH was determined after 7 days.

### Statistical analyses

Statistica 6.0 was used for designing of the Taguchi experiment. The results were analyzed using factorial ANOVA of the design module in Statistica 6.0 to determine which factor was significant in affecting the percentage and rate of Flu and Phe biodegradation.

## Results

### Identification of microbial strains

Six gram-negative bacterial strains were isolated from enrichment consortium (SBU) on nutrient agar plates. Among the isolated strains (SBU1- SBU6) only SBU1 showed successfully grown on PAHs MSM plate and turned the color of the plate to the red color after 7 days. Isolated strains were first identified using biochemical tests. Further molecular identification of bacterial strains was performed by amplification and sequencing the 16S rDNA and comparing them to the database (Table 3).

**Table 3:** **Identification of bacterial isolates by 16S rDNA and their growth on Flu and Phe as the sole source of carbon**

**Isolate**	**Closest hit**	**Accession numbers**	**Flu**	**Phe**	**Flu + Tween-80**	**Phe + Tween-80**
SBU1	*Roseovarius pacificus*	KF052989	9.2	9.9	9.6	14.3
SBU2	*Marinobacter hydrocarbonoclasticus*	KF052990	2.6	10.6	20.6	26.0
SBU3	*Pseudidiomarina sediminum*	KF052991	0.0	1.5	0.0	7.8
SBU4	Unidentified	-	5.1	3.7	6.4	15.5
SBU5	Unidentified	-	5.3	8.9	3.0	3.8
SBU6	Unidentified	-	0.0	8.6	9.7	11.0
Mix of isolated strains	-	-	16.7	21.4	21.3	24.1
Enrichment consortium (SBU)	-	-	64.0	58.4	59.9	40.8

### Biodegradation of Flu and Phe by enriched consortium and bacterial isolates

The biodegradation ability of each isolated strain, as well as collaborative efficiency of all six strains and the enriched consortium considering two scenarios: first, by using 1 w/w% Tween-80 as a surfactant; second, by using surfactant free media are presented in Table 3. In general, the abiotic losses of Flu and Phe during the 7-day biodegradation experiments were negligible and all isolated strains had poor degradation abilities (<20% of the Flu and Phe degradation after 7 days). When isolated strains were mixed together, higher Flu and Phe degradation rates were achieved reaching to 16.7% and 21.4% for Flu and Phe when Tween-80 was not added and 21.3% and 24.1% in the presence of Tween-80, respectively. SBU consortium had highest degradation percentages (Table 3).

### Biodegradation optimization of Flu and Phe by enriched consortium

**Figure 1:**
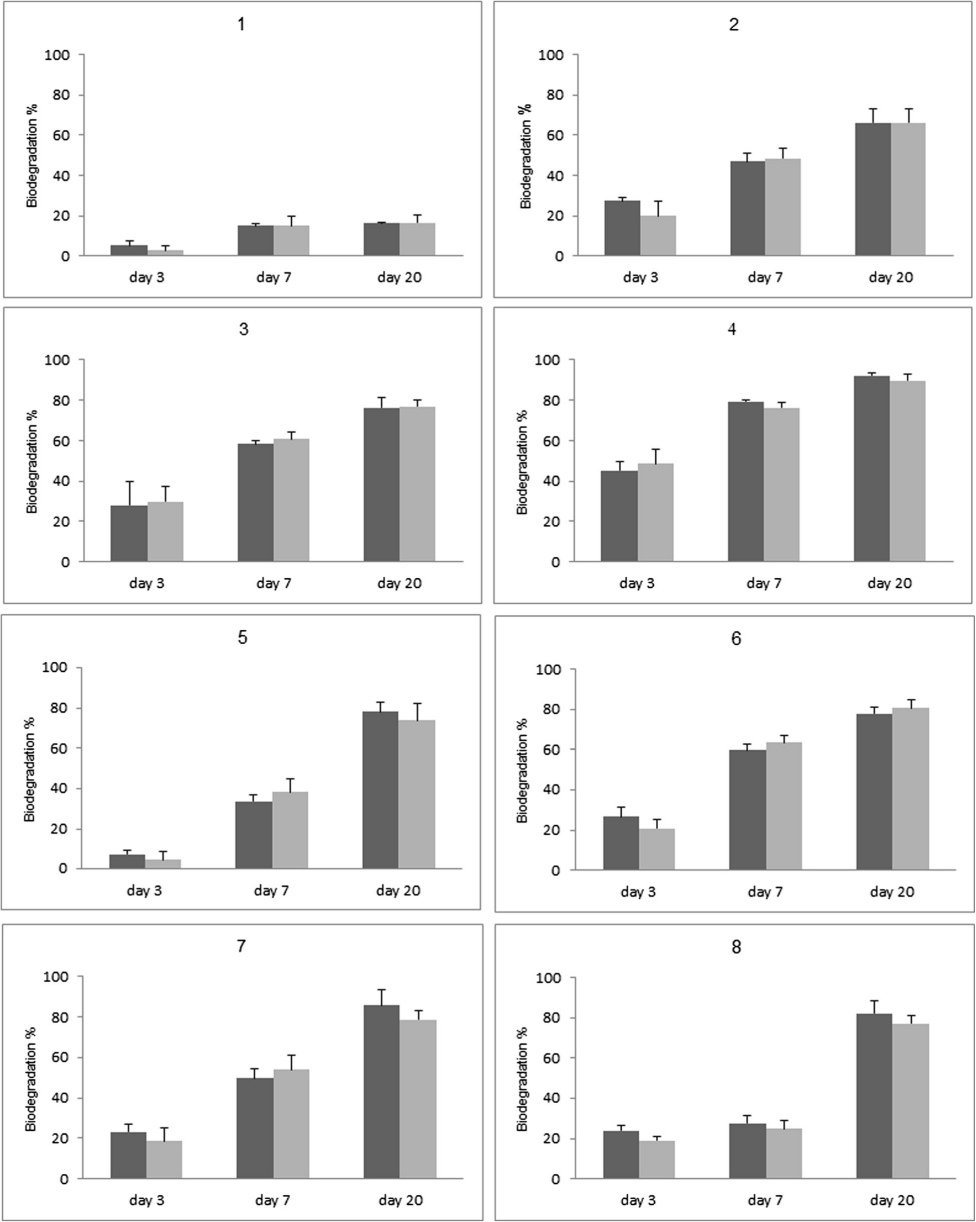
**Concentrations of residual PAHs (trials 1-8) during 20 days experiment with the inoculation of enriched consortium (Flu: dark bar, Phe: light bar).**

**Figure 2:**
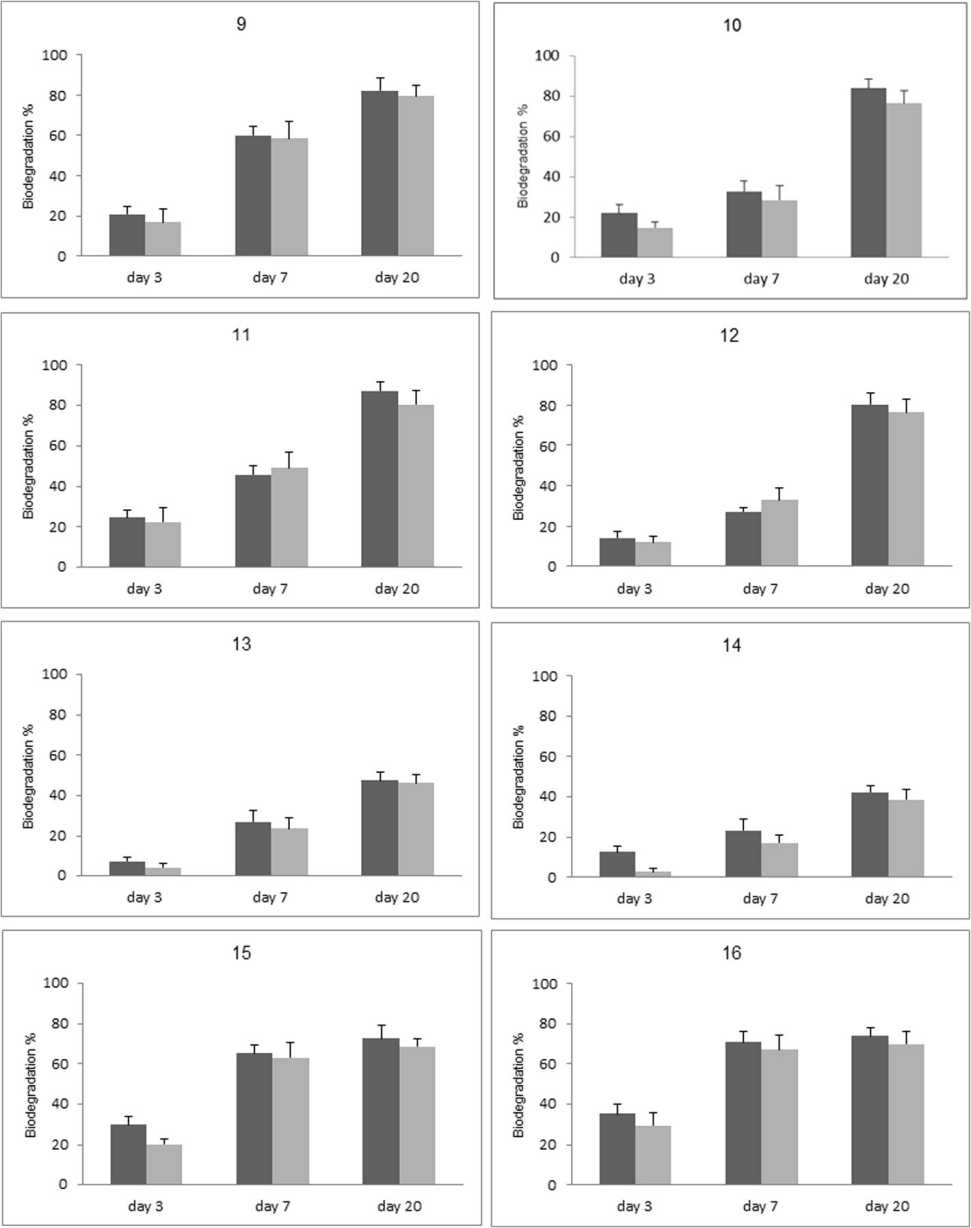
**Concentrations of residual PAHs (trials 9-16) during 20 days experiment with the inoculation of enriched consortium (Flu: dark bar, Phe: light bar).**

During the Taguchi experiment, the changes in Flu and Phe concentrations in control trials varied between 5 and 29%. Concentrations of residual PAHs during 20 days experiment are shown in Figure 1. Achieved biodegradation rates of Flu and Phe were fitted by the first order rate model with R_2_ values > 0.9 (Table 2) except in trial numbers 1 (Figure 1), 15, and 16 (Figure 2) which also had the lowest Flu and Phe biodegradation. The degradation percentages and degradation rates (k) in each trial is summarized in Table 2. The highest Flu and Phe biodegradation percentage at the end of 20^th^ day and also the largest first order rate constant were achieved in trial 4 as follows:

1. Degradation percentages (%) Flu = 92.18 ± 1.51; degradation rate (k) Flu = 0.122
2. Degradation percentages (%) Phe = 89.84 ± 3.03; degradation rate (k) Phe = 0.108

On the other hand, the least amount of Flu and Phe biodegradation percentages and first order rate constants were found in trial 1 (Table 2).

### Factors affecting biodegradation of phenanthrene and optimal condition

The ANOVA results of S/N ratio comparisons based on the values of the first order rate constants (k) and biodegradation percentages at the end of the 20-day experiment are shown in Table 4. In general, all 5 tested factors had significant effect (p < 0.05) on the biodegradation percentages and the k values for both Flu and Phe, with the inoculum size and pH as the most effective factors in Flu and Phe biodegradation and first order constant (k) respectively (based on the calculated effect sizes) (Table 4). Theoretically optimized levels for considered factors based on 20-day biodegradation process results or first order constant (k) values were predicted as follows:

**Table 4:** **Results of S/N ratio comparisons based on the values of the first order rate constants (k) and biodegradation percentages at the end of the 20-day experiment**

	**Final biodegradation (%) Flu**			**First order rate constant (k) Flu**			**Final biodegradation (%) Phe**			**First order rate constant (k) Phe**		
**Factors**	**F**	**p-value**	**Effect size**	**F**	**p-value**	**Effect size**	**F**	**p-value**	**Effect size**	**F**	**p-value**	**Effect size**
pH	72.93	0.00	0.0144	27.16	0.00	0.0214	52.69	0.00	9.70	21.52	0.00	11.86
Temperature	70.98	0.00	0.0145	19.20	0.00	0.0178	53.04	0.00	9.80	14.54	0.00	10.66
Inoculum	58.79	0.00	0.0165	9.05	0.00	0.0138	57.99	0.00	11.09	15.34	0.00	9.66
Salinity	15.91	0.00	0.0092	7.37	0.00	0.0161	9.98	0.00	4.36	3.67	0.02	5.73
C/N	16.24	0.00	0.0082	5.49	0.00	0.0118	15.50	0.00	4.98	4.79	0.00	5.48

1. 20-day biodegradation process _(Phe & Flu)_: pH = 7; temperature = 35°C; inoculum size_OD600_ = 0.4; salinity = 40 ppt & C/N = 100/40
2. First order constant (K) _(Phe & Flu)_: pH = 7; temperature = 35°C; inoculum size_OD600_ = 0.4; salinity = 40 ppt & C/N = 100/10

**Figure 3:**
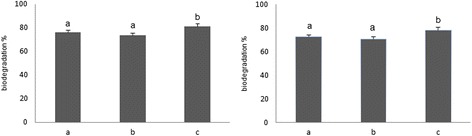
**Comparison of Flu (right) and Phe (left) biodegradation efficiency between predicted optimized conditions based on the values of the biodegradation percentages (a) or first order rate constants (b) and Taguchi trial 4 (c).**

Nevertheless, when practical experimental trials based on the above optimized conditions were conducted and compared with the trial 4 (the one with best practical result) both optimized conditions resulted in significantly weaker biodegradation efficiency (Figure 3).

## Discussion

In our study the six bacterial strains isolated from mangrove surface aerobic sediments- Nayband Bay, Iran included *Marinobacter hydrocarbonoclasticus*, *Roseovarius pacificus*, *Pseudidiomarina sediminum* and 3 unidentified strains. PAHs degrading activities of the bacterial consortium belonging to the genus *Marinobacter* [13,14], and R*oseovarius* [15,16] have been reported worldwide. In addition, Darmayati et al*.* [17] isolated *Pseudidomarina sp.* from oil polluted marine sediments but no PAH degrading abilities were reported previously. Also, *Roseovarius pacificus* has been isolated from a polycyclic aromatic hydrocarbon (PAH) degrading consortium-deep sea waters of the Indian Ocean, but there is no literature concerning the biodegradation of PAHs by *Marinobacter hydrocarbonoclasticus* and *Pseudidiomarina sediminum*.

As confirmed by previous studies [18] addition of Tween-80 to the culture media as a surfactant resulted in increased biodegradation of Flu and Phe by nearly all strains in our study. However addition of Tween-80 had negative effects on biodegradation ability of the SBU5 strain which could be due to strain specific parameters including different toxicity levels of a surfactant and/or competitive utilization of surfactant and PAHs [19,20].

Our enriched consortium performed higher Flu and Phe biodegradation percentage compared with a single strain cultures or all-strain mixture cultures. This could be because of an inappropriate isolation or mixing. On the other hand, several studies have shown that biodegradation caused by mixed culture is more effective than those circumstances when pure cultures are used which may be due to a broader enzymatic capability and counteraction of toxic intermediates by co-metabolic processes [21,22]. Also, microbial biodegradation efficiency has been found to depend on other factors. In essence, assessment of the optimal conditions would make the bioremediation process more effective.

In our study, when predicted optimal conditions suggested by models based on biodegradation percentages or first order rate constant (k) were tried, equal removal efficiency for both Phe and Flu were achieved. Interestingly, the biodegradation percentage of trial 4 in Taguchi design was significantly higher than predicted optimum conditions. Given that, only the pH was different between trial 4 and predicted optimized conditions, practically optimized levels for pH, inoculums size, salinity, temperature, and C/N ratio were adjusted to 8, 0.4, 35°C, and 100/40 respectively.

Among environmental variables temperature is one of the most important factors affecting biodegradation of petroleum hydrocarbons [23]. In the current study, the best biodegradation efficiency was achieved when the highest temperature (35°C) was retained which was also near to the natural conditions where samples were collected. This could be due to the increased solubility of PAHs at higher temperature causing a noticeable improvement in the bioavailability of Flu and Phe molecules [24,25]. Also bacterial metabolism may increases as with temperature increase [26].

In terms of salinity, our results proposed 40 ppt salinity as optimum level for Flu and Phe biodegradation which was similar to those levels measured in the Nayband Bay. This may be due to the adaptation of bacteria to environmental conditions. Shiaris [23] found positive correlation between salinity and rates of biodegradation of Phe and naphthalene in estuarine sediment but some researchers reported decreased rates of hydrocarbon metabolism when salinity was increased and concluded that this may be because of negative effects of ions on metabolic rates of bacterial cells [27].

The pH also has been found to have an important effect on biodegradation. Culture medium pH can affect microbial diversity and activity possibly through altering the enzymatic activity, transport processes and the nutrient solubility [27]. The biodegradation process was found to be active throughout the pH = 6-8 range in the current study and More than 70% of Flu and Phe was degraded when the pH of culture medium ranged from 7 to 8, but the biodegradation percentage was lower in pH 6.5 (about 55%). Leahy and Colwell [26] have reported that most petroleum degrading bacterial species can perform degrading action at pH = 6–8, but the optimum degradation abilities occur near pH = 7. Inorganic nutrients have been also suggested to play a key role in the process of PAHs biodegradation and low inorganic nutrient supply has been reported to limit the efficiency of PAH biodegradation in marine environment [28]. The present study supports the above mentioned phenomena by providing evidences for enhanced effects of N fertilizers on bacterial growth and biodegradation percentages. The molar C/N ratio of 100/10 is a generally recommended condition for PAH degradation (based on the average values of elemental microbial cell composition and general microbial crude cellular extract) [11] which is also convenient with our biodegradation percent based predicted model results. However, the C/N ratio for the Taguchi trial 4 as well as first order rate constant (K) based predicted value was 100:40 (molar). Meanwhile, no significant difference was found between biodegradation percentages of both predicted scenario. In this case, supplying the Taguchi trial 4 with 100:10 C/N may also reveal the same result as the 100:40 ratio which can be considered in industrial applications. Finally, the estimated optimum inoculum size was large which may be due to a longer lag phases for smaller inoculums concentrations.

## Conclusion

Our results showed that, indigenous bacteria from mangrove surface sediments of Nayband Bay have high potential to degrade Flu and Phe with the best results achieved when enriched consortium- SBU was used. It can be speculated that although mathematical optimization can improve the biodegradation efficiency of SBU, practically efficient applications may be made with different conditions. Also, successful biodegradation efficiency of a single strain or mixtures may be accomplished during the process of optimization.
